# Femur First navigation can reduce impingement severity compared to traditional free hand total hip arthroplasty

**DOI:** 10.1038/s41598-017-07644-4

**Published:** 2017-08-03

**Authors:** Arnab Palit, Mark A. Williams, Glen A. Turley, Tobias Renkawitz, Markus Weber

**Affiliations:** 1 WMG, University of Warwick, Coventry, CV4 7AL UK; 2 Department of Orthopedic Surgery, Regensburg University, Medical Center, 93077 Bad Abbach, Germany

## Abstract

Impingement is a major source of dislocation and aseptic loosening in total hip arthroplasty (THA). We compared impingement free range of motion (ROM) using a novel computer navigated femur first approach to conventional THA. In addition, impingement between genders was also explored. In a retrospective analysis of 121 THA patients, subject-specific post-operative ROM was simulated using post-operative 3D-CT data, and compared with the benchmark ROM, essential for activities of daily living. Three parameters were defined to express both implant-to-implant (ITI) and bone-to-bone (BTB) impingement - coverage percentage, third angle, and impingement severity. Although coverage percentage was similar between the navigated and conventional group for both ITI (p = 0.69) and BTB (p = 0.82) impingement, third angle was significantly reduced in the navigation group for both ITI (p = 0.02) and BTB (p = 0.05) impingement. Impingement severity for both ITI (p = 0.01) and BTB (p = 0.05) was significantly decreased in the navigation group compared to the conventional. Impingement severity in men was considerably higher compared to women for both ITI (p = 0.002) and BTB (p = 0.02). Navigation guided femur first THA is able to improve alignment of ROM axis, and consequently, to reduce impingement in THA. Men seem to be more prone to impingement than women.

## Introduction

Total hip arthroplasty (THA) is considered as one of the most effective techniques to restore lost manoeuvrability to patients suffering from osteoarthritis (OA), acute trauma and rheumatoid arthritis (RA)^1, 2^. One of the key intraoperative challenges while performing THA is to find an optimised compromise amongst hip biomechanics, tribology, and post-surgery functionality. Orientation of the prosthetic components is one of the critical factors during THA in order to achieve stable joint and ideal range of motion (ROM) so that the patient could accomplish their activities of daily living (ADLs). Component mal-positioning and soft tissue imbalance would lead to two of the most significant reasons for revision surgery - (a) aseptic loosening, and (b) dislocation^3–6^. It was found that 90% of dislocations had evidence of impingement^7^. Impingement can be caused due to—(a) component-to-component contact (prosthetic impingement), (b) component-to-bone contact (bone-to-prosthesis impingement, or (c) bone-to-bone contact (bony impingement)^5^. Impingement in THA results greater component wear, limited range of movement (ROM) with reduced hip functionalities, and increased pain^8, 9^. Additional movement beyond the impingement point leads to subluxation of the femoral head until the joint dislocates^3, 4, 10^. Thus, improved range of motion to impingement would directly improve resistance to dislocation and wear^11^.

Two new developments for THA could potentially combat and address these complications. Firstly, an innovative computer-assisted THA operation, which implemented the concept of ‘femur first’/ ‘combined anteversion’ was introduced to overcome the limitations of the traditional THA^5, 12^. This approach combined several aspects in performing a functional optimisation of the cup position, and extensively addressed ROM while maintaining cup alignment and containment parameters as detailed by Renkawitz, *et al*.^5^. Secondly, a comprehensive ROM benchmark of hip joint for the ADLs was established by Turley, *et al*.^13^. This ROM benchmark is a powerful tool to graphically represent the loss of mobility, and therefore, could be used in measuring the effectiveness of THA operation by comparing the post-operative simulated ROM with the required benchmark ROM.

Therefore, two research objectives were addressed in the current study.

- To assess whether a computer navigated femur first approach is more effective with regard to providing an impingement free range of motion in comparison to conventional THA as measured by ROM size, ROM alignment and impingement severity of THA patients in a virtual range of motion simulation using post-operative CT scan data
- To assess whether there are sex specific differences in ROM as measured by ROM size, ROM alignment and impingement severity

## Materials and Methods

### Brief Overview of Surgical Procedures

In this study, the effectiveness of newly introduced navigated minimally invasive THA surgical procedure was investigated compared to the conventional minimally invasive THA. The retrospective (secondary) analysis, detailed in this paper, expanded the previous published results^12^ and now focused on a detailed impingement investigation using a different methodology. Instead of measuring single directional movements (for example only flexion or only rotation), the current analysis combined all the directional movements to provide a detailed 3D range of motion (ROM) analysis. As a result, several parameters such as ROM area, ROM alignment and severity of impingement, which are related to measure the effectiveness of the surgical outcome, were explored in this study. A brief description of the navigated and conventional surgical produce is presented below.

- Conventional minimally invasive THA (CTHA)
  In conventional minimally invasive THA, acetabular components were placed in a ‘safe zone’ without using any alignment guides. The ‘safe zone’ was defined by Lewinnek, *et al*.^14^ (inclination = 40° ± 10°, and anteversion = 15° ± 10°), and this hypothetical ‘safe zone’ was estimated visually by the surgeon during operation. The femoral cementless components were implanted in a best fitting position according to the three dimensional (3D) geometry following the natural bow of the femoral canal.
- Navigated minimally invasive ‘femur first’ THA (NTHA)

**Figure 1:**
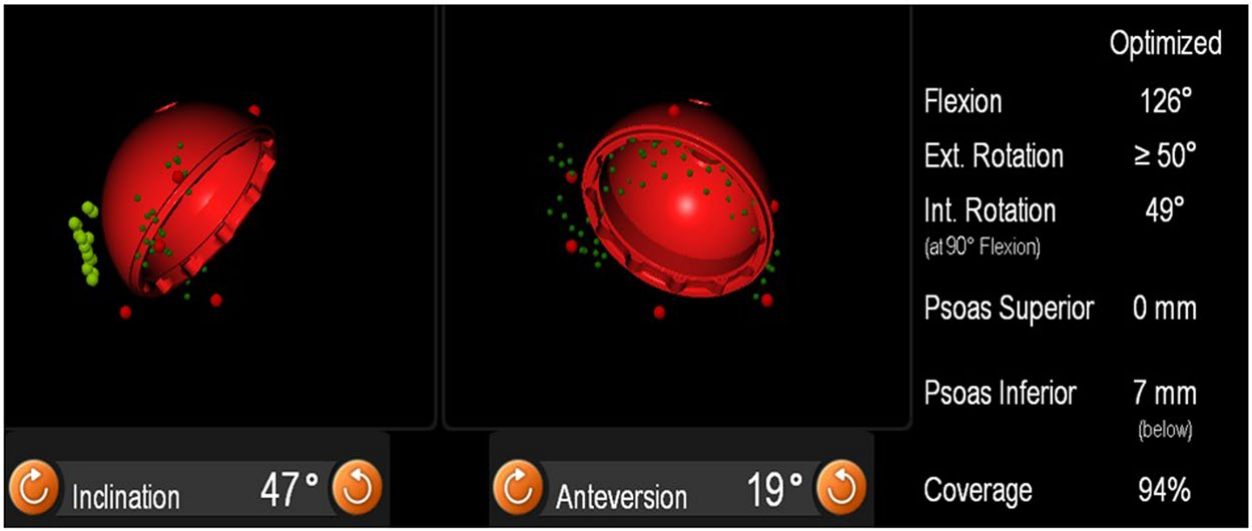
Optimized cup position as calculated by an intraoperative impingement detection algorithm using imageless navigation.

An imageless navigation system (Hip 6.0 prototype, Brainlab, Feldkirchen, Germany) along with a ‘femur first’ prototype software were used to perform navigated minimally invasive ‘femur first’ THA. The registration process for navigated THA in a lateral decubitus position and the measurement of stem anteversion were carried out by following the procedure described in Sendtner, *et al*.^15^, Renkawitz, *et al*.^5^, Turley, *et al*.^6^ and Renkawitz, *et al*.^12^. Anterior pelvic plane was defined by left anterior superior iliac spines, right anterior superior iliac spines, left pubic tubercles and right pubic tubercles. These four locations were registered using a reference pointer which was located on the surface of the skin. The medial and lateral epicondyles and ankle points were also registered for femur^16^. Thereafter, the anatomy of acetabular was registered and reamed. Based on the information collected during the preparation of the femur and acetabulum, the navigation system calculated the optimised position of acetabular component for impingement free manoeuvre of hip joint. This information was presented to the surgeon on a screen (Fig. 1). Guided by the three-dimensional (3D) projections on the navigation screen, the acetabular component was inserted, followed by insertion of the uncemented femoral component. A detailed description of the navigated THA was presented in Renkawitz, *et al*.^5^ and Renkawitz, *et al*.^12^.

### Design of the Study

The current study is a secondary outcome analysis of data obtained in a patient as well as an observer-blinded randomized controlled trial (RCT) approved by the local medical ethics committee (10-121-0263). CT scan of pelvic and femur, required to assess post-operative condition after six week (approximately), was approved by German Federal Office for Radiation Protection. RCT was registered at the German Clinical Trials Register with a Main ID DRKS00000739. Design of the study including sample size, randomisation, and recruitment of patients, exclusion, and inclusion criteria are detailed in Renkawitz, *et al*.^5^ and Renkawitz, *et al*.^12^.

In brief, a series of 783 patients, who were admitted for primary uncemented THA due to primary or secondary osteoarthritis at our institution in between December 2011 to March 2013, was screened. According to the protocol of the main study^17, 18^, eligible participants were mainly selected based on two conditions: (a) the age of the patients should be in between 50 to 75 years, and (b) the American Society of Anesthesiologists (ASA) score should be 3 or below for the patients who were admitted for primary cementless unilateral THA attributable to primary or secondary osteoarthritis. In addition to these conditions, the patients were not selected if they had arthritis due to hip dysplasia, post-traumatic hip deformities, and/or a previous hip surgery.

To allow gait analysis, as intended for the primary outcome, only those patients were included who had no significant disease of the contralateral hip. Due to these strict inclusion criteria, out of 783 screened patients, 597 did not meet the inclusion criteria. Twenty-seven (27) patients declined to participate and 19 patients had to be excluded for other reasons (e.g. cancellation of the operation due to elevated inflammatory markers). The first five navigated cases were regarded as learning curve. Finally, 135 patients were randomised to either navigated or conventional THA after informed consent had been obtained. A detailed description of the randomisation algorithm was included in^12^.

THA was performed with all patients in the lateral decubitus position using a minimally invasive single-incision anterolateral approach by four experienced orthopaedic surgeons (JG, ES, MW, TR). Each surgeon had experience with more than 200 conventional and navigation-controlled THAs. Press-fit acetabular components and cement-free hydroxyapatite-coated stems (Pinnacle®cup, Corail®stem; DePuy, Warsaw, IN, USA) with metal heads of 32 mm were used. Cup diameter was chosen according to the natural geometric configuration of the acetabulum. No cups below size 48 were used to enable combination with 32 mm heads. Out of the initially 135 randomized patients, five (3.7%, four in the navigated and one in the control group) did not receive the allocated intervention. Of these five one case with shut down of the navigation system was included in analysis per intention to treat (ITT). Another eight patients (5.9%, six in the navigated and two in the control group) had to be excluded from analysis due to missed or incorrect CT (four patients) or withdrawn informed consent (four patients). In contrast to the primary study analysis, two additional CT data sets (one in the navigated and one in the control group each) were not compatible with the novel three dimensional impingement analysis. Altogether 121 data sets were included for final analysis. Anthropometric characteristics of the navigation and control group as well as intraoperative data were comparable (Table 1). All the methods were carried out in accordance with the relevant guidelines and regulations.

**Table 1:** Patient characteristics and intraoperative data in this study.

Characteristic	Conventional (n = 65)	Navigated (n = 56)
Sex (female/male)	32/33	32/24
Age (years)	62.6 ± 7.9	62.8 ± 7.4
BMI (kg/m^2^)	27.2 ± 4.3	27.0 ± 4.0
Treatment Side (left/right)	27/38	29/27
ASA I	16	8
ASA II	30	33
ASA III	19	15
Kellgren Score	9 (5–10)	8 (6–10)
Length of skin incision (cm)	10.3 ± 1.2	10.5 ± 1.2
Operating time (minutes)	64.4 ± 14.0	71.3 ± 12.3
Cup size	54 (48–60)	54 (50–62)
Stem size	12 (9–15)	12 (10–16)
Cup inclination (°)	42.3 ± 6.4	42.5 ± 5.2
Cup anteversion (°)	17.5 ± 9.0	18.3 ± 6.9
Stem anteversion (°)	7.0 ± 8.8	9.1 ± 10.4
Cup coverage (%)	87.7 ± 9.6	87.4 ± 9.0

### Modelling Benchmark ROM

**Figure 2:**
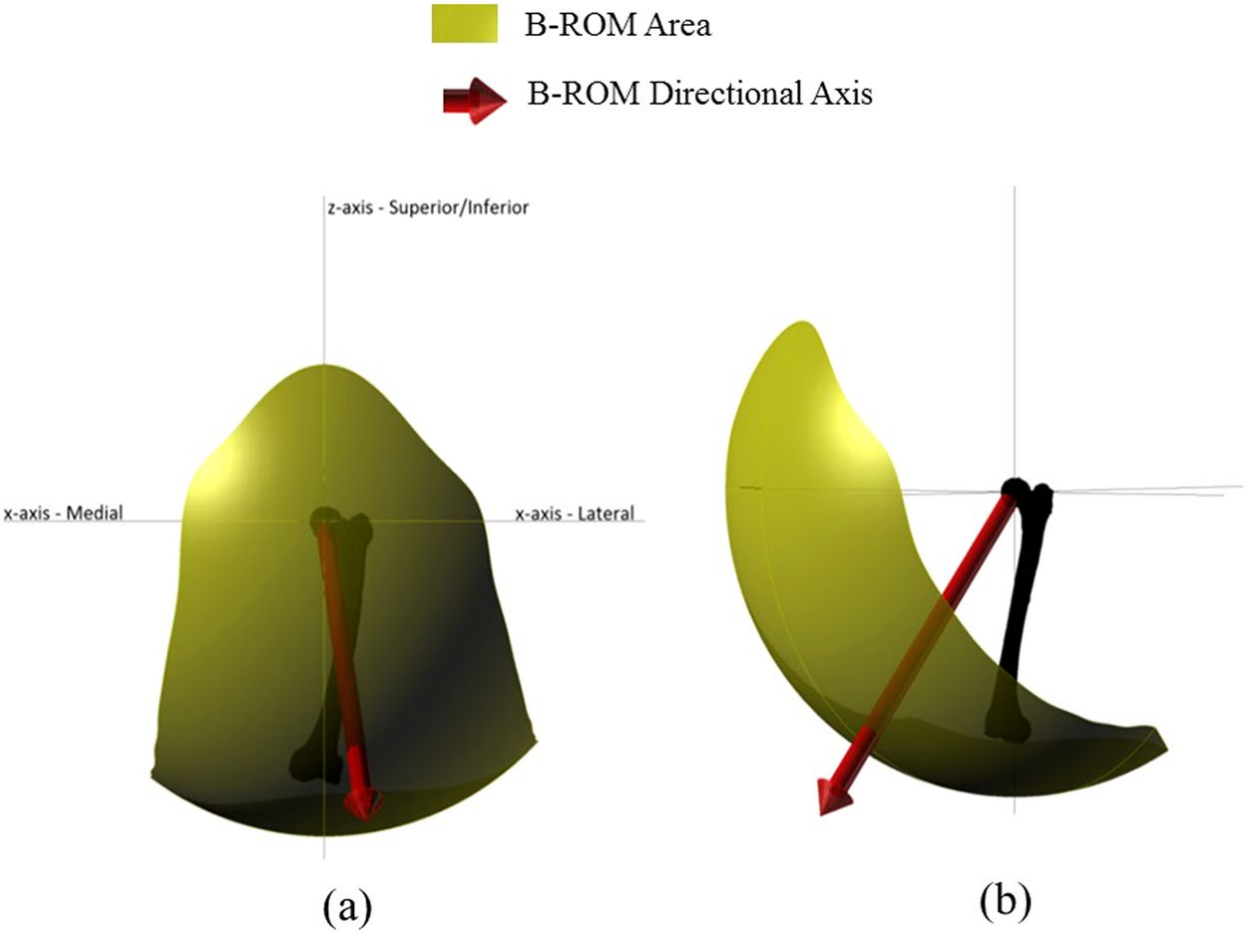
Representation of the ROM benchmark area and corresponding directional axis of ROM area. (**a**) Coronal view, (**b**) Sagittal view.

A ROM benchmark, developed by Turley, *et al*.^13^, Turley^17^, was used in order to evaluate the post-operative hip joint motion, and consequently, the effectiveness of surgical operation. The data used to construct this ROM benchmark boundary was based on the mean reference values for (a) pure joint motion, and (b) fifteen activities of daily living (ADLs) motion, collected through systematic literature review^13^. The ROM benchmark boundary was characterised by two attributes– (a) its area and shape which is spherical in nature (Fig. 2a and b) its position relative to the anatomical coordinate system (Fig. 2b). The position is defined using a directional axis (Fig. 2b). Detailed description of the methodology to calculate ROM area and directional axis was included in Turley, *et al*.^13^.

### Modelling Subject-Specific Post-Operative ROM

**Figure 3:**
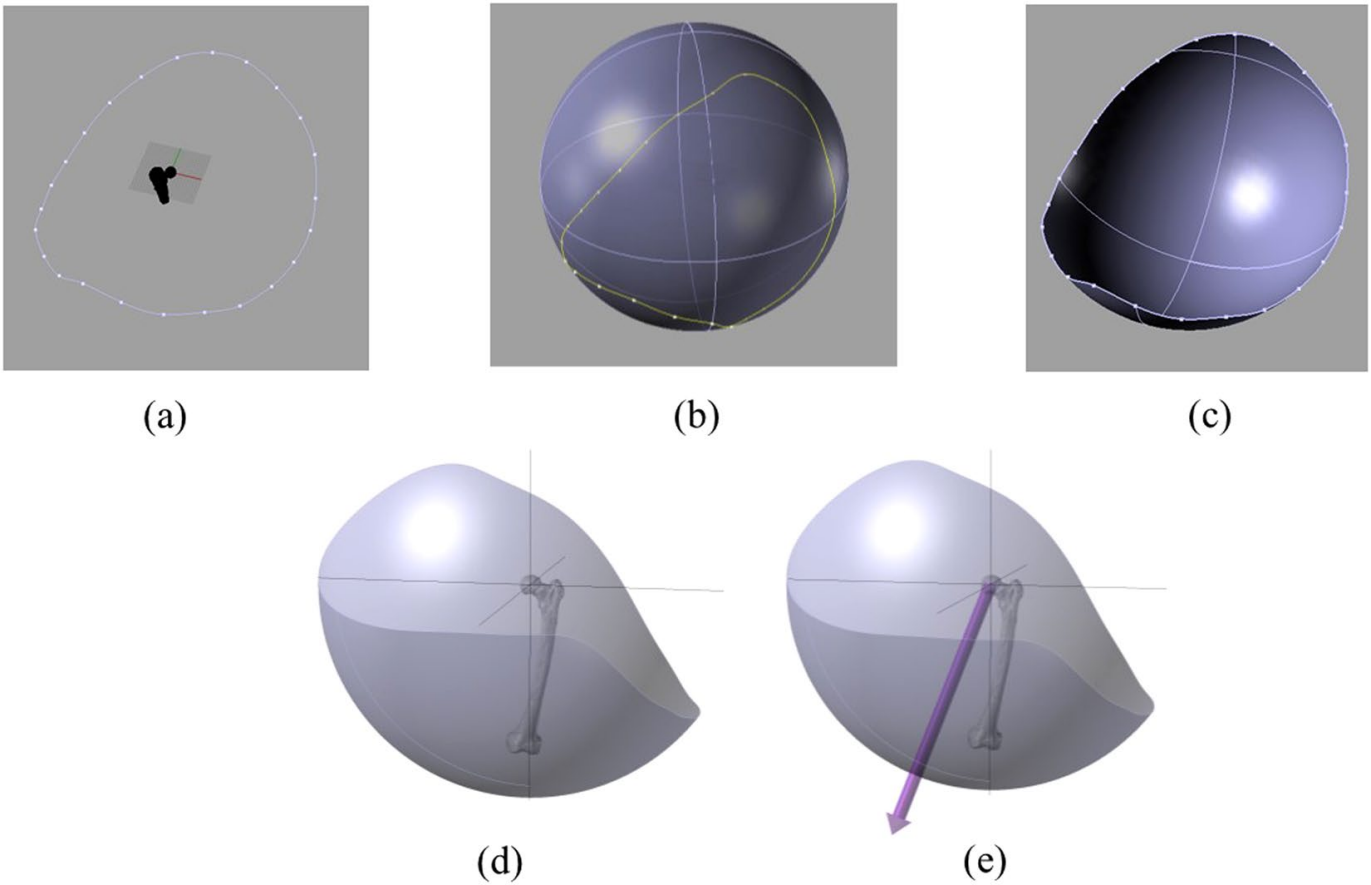
Schematic representation of the modelling procedure to construct subject-specific post-operative ROM area. (**a**) A set of collision points represents ROM boundary, (**b**) A sphere containing the ROM boundary. Centre of the sphere is the centre of rotation of ROM simulation and radius is the distance between the centres of rotation to the knee centre. (**c**) ROM area. (**d**) ROM area in another view. (**e**) Directional axis for prosthetic ROM area.

Post-operative pelvic and femoral CT scans were performed after five to seven weeks of surgery (Somatom Sensation 16; Siemens, Erlangen, Germany). Segmentation and construction of the subject-specific pelvic bone, femur, and prosthetic components (the metal acetabular and femoral components) were carried out by an independent external institute (MeVis Medical Solutions, Bremen, Germany), blinded to individual patient data and the type of surgical approaches. To align the prosthetic components with the bones, the reference measurements captured by the navigational system during the surgical procedure were utilised. Similar post-operative measurements were also taken from the group, which received non-navigated procedure during the controlled trial. The reference measurement points were imported into Rhino (Robert McNeel & Associates, US) along with the bone and implant geometries. These imported points were used to define pelvic and femoral coordinate frames, which were then utilised to align the implants accordingly. Thereafter, a rhino script was developed which simulated the femoral movement until a collision occurred between the components or bones following the rotation through the medial-lateral axis. With the femur returned to the centre position again, it was rotated until collision occurred, and the procedure continued through an increment of 15° in the transverse plane. When collision occurred, the position of knee centre point was recorded. Therefore, a set of collision points were collected through 360° rotation of femur around medial-lateral axis (Fig. 3a). The ROM boundary was then established by interpolating a line through the simulated points (Fig. 3a). This methodology was developed by Turley^17^. A sphere was then constructed whose centre was the centre of rotation and the radius was the distance from the centre of rotation to knee centre (Fig. 3b). Thereafter, the sphere was cut using ROM boundary to define ROM surface area as shown in Fig. 3c and d.

The position of the prosthetic ROM surface with respect to the anatomical coordinate system was defined using a directional axis (Fig. 3e). In order to calculate this directional axis (Fig. 3e), moment of inertia technique^18, 19^ was used by constructing a best fit plane from ROM boundary points, as identified through simulation. A detailed description of this method was explained in Turley^17^ and Turley, *et al*.^6^.

### Measuring Impingement

The component collision was classified into two different groups, Implant-To-Implant (ITI) contact and Bone-To-Bone (BTB) contact. During ITI contact, only the cup implants and the femur implants were considered to simulate their interaction. This instance could be used to decide initial cup and femoral positioning. For BTB, the simulation used all the relevant components including the bony structures, which were the pelvis, the pelvis implants, the femur, and the femur implants, to model their interaction with each other. The latter case is fully representative of the patient’s full ROM. Therefore, the subject-specific post-operative ROM (PO-ROM) area was calculated for both ITI and BTB cases – (a) PO-ROM for ITI, and (b) PO-ROM for BTB. The coverage percentage was defined by the ratio of calculated PO-ROM area and benchmark ROM (B-ROM) as defined by equation (1).

1 $$Coverage\,Percentage\,=\,\frac{Post\,Operative\,ROM}{Benchmark\,ROM}\times 100$$

When the coverage percentage is above 100%, the ROM area is large enough to cover the benchmark ROM area. However, impingement can still be occurred in these cases if the area is poorly located. Thus, the orientation and positioning of PO-ROM area was defined relative to the benchmark ROM by an angle, termed as ‘third angle’ (Fig. 3e). It was a three dimensional (3D) angle between the directional axis of the postoperative (PO-ROM) and the directional axis of the benchmark ROM (B-ROM). The lower the 3D angle, the better is the match between the PO-ROM axis and the B-ROM axis.

**Figure 4:**
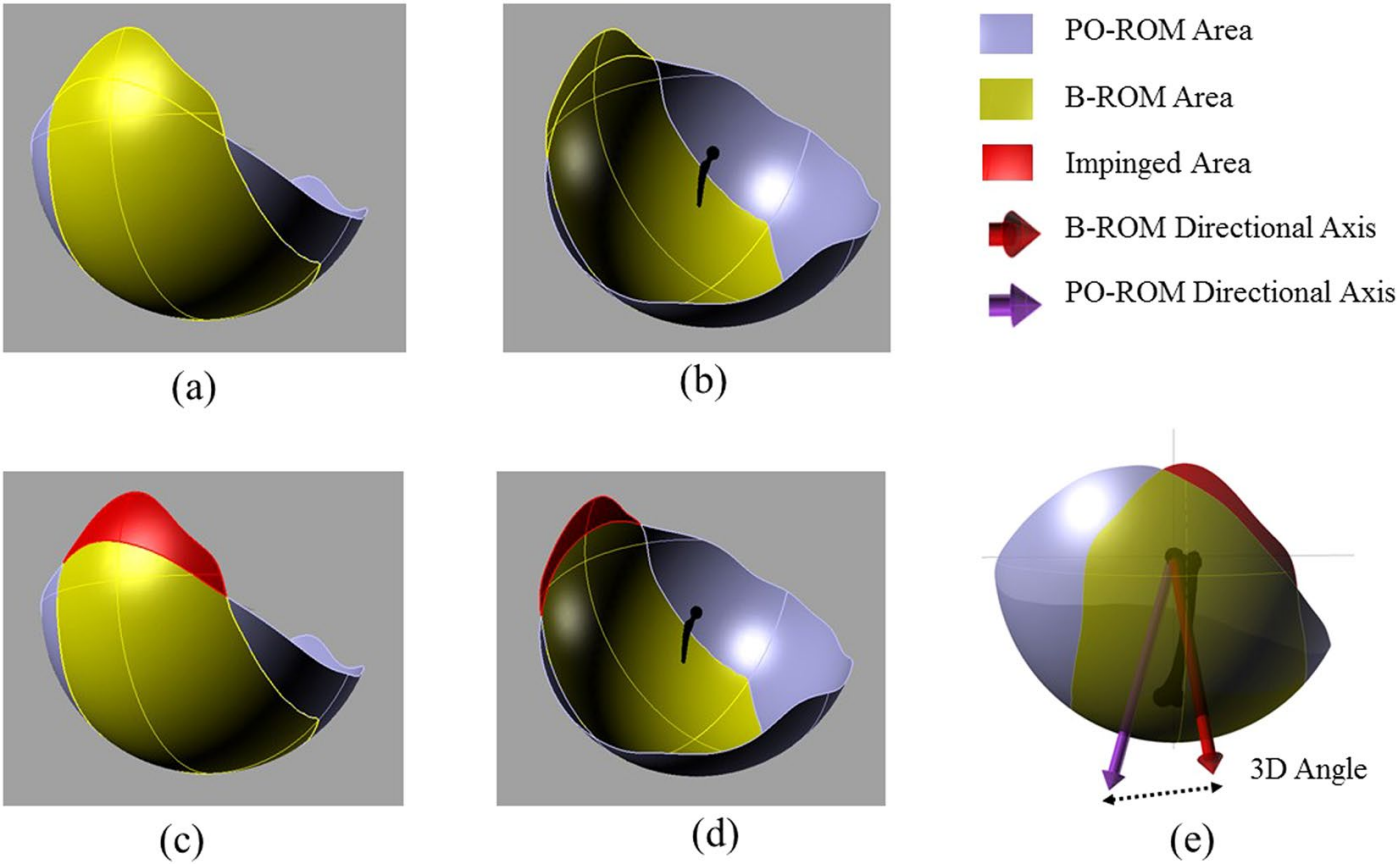
Schematic representation of PO-ROM area, B-ROM area, Impingement area, and 3D angle. (**a**) and (**b**) B-ROM overlaid PO-ROM; (**c**) and (**d**) Impingement area which is the part of BO-ROM area not covered by PO-ROM area; (**e**) Difference between BO-ROM directional axis and PO-ROM directional axis in terms of 3D angle.

Impingement severity was calculated by comparing the subject-specific post-operative ROM (PO-ROM) area with the benchmark ROM (B-ROM) area (equation 2). The impingement would occur if the B-ROM area was not covered by the PO-ROM area (Fig. 4a,b). Therefore, the impingement area (Fig. 3c,d) was defined by the fraction of the B-ROM area which was not covered by the PO-ROM area. Therefore, impingement severity (IS) was defined as follows

2 $$Impingement\,severity\,(IS)\,=\,\frac{Impingement\,{\rm{Area}}}{Benchamrk\,ROM\,Area}\,\times \,100$$

Impingement severity is calculated for both ITI and BTB impingements. The severity increased with the increase of IS values. When there was no impingement, IS = 0, and therefore, severity is minimum.

### Statistical Analysis

Statistical analyses were performed using SPSS v22.0.0 (IBM, Armonk, New York). Statistical significance was defined as a p-value < 0.05. The data between the two treatment groups (i.e. navigated and conventional surgical procedures) were compared by the Mann-Whitney U test (for continuous variables).

## Results

**Figure 5:**
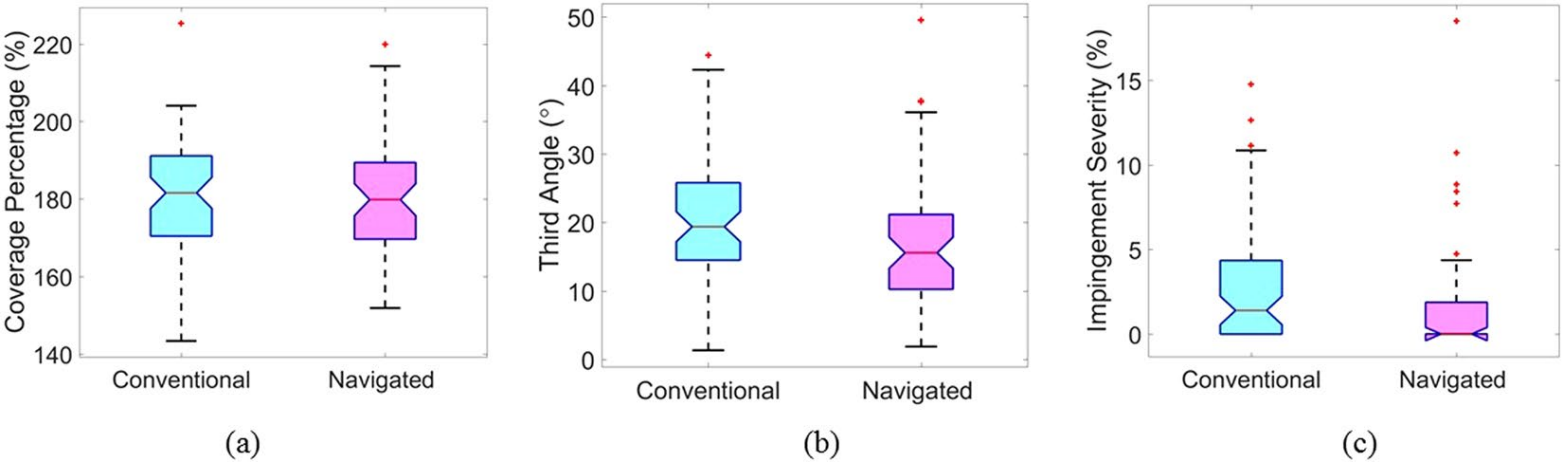
Box plot of (**a**) Coverage percentage, (**b**) third angle, (**c**) impingement severity for ITI impingement.

Analysing ROM area for ITI impingement, it was found that there is no significant difference (p = 0.69) in coverage percentage between the navigated group with (M = 179.9%, SD = 14.4%, Fig. 5a) and the conventional group with (M = 180.7%, SD = 14.5%, Fig. 5a). However, PO-ROM axis for ITI impingement as measured by third angle was significantly closer (p = 0.02) to the B-ROM axis in the navigated group (M = 17.0°, SD = 9.9°, Fig. 5b) compared to the conventional group (M = 20.1° SD = 9.1°, Fig. 5b). In addition, impingement severity for ITI impingement was significantly decreased (p = 0.01) in the navigation group (M = 1.6%, SD = 3.4%) in comparison with the control group (M = 2.6%, SD = 3.4%, Fig. 5c).

**Figure 6:**
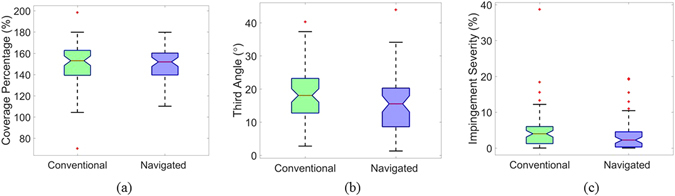
Box plot of (**a**) Coverage percentage, (**b**) third angle, (**c**) impingement severity for BTB impingement.

ROM analysis for BTB impingement showed similar results to ITI values. Coverage percentage for BTB impingement was not statistically significant (p = 0.82) between the navigated group with (M = 151.0%, SD = 15.5%, Fig. 6a) and the conventional group with (M = 150.6%, SD = 20.3%, Fig. 6a). However, it was observed that the PO-ROM alignment with respect to B-ROM directional axis for BTB impingement as measured by third angle was statistically improved (p = 0.05) in navigated group (M = 15.5°, SD = 8.1°, Fig. 6b) in comparison with the conventional group (M = 18.3°, SD = 7.7°, Fig. 6b)). Consequently, impingement severity for BTB impingement was significantly reduced (p = 0.05) in the navigated group (M = 3.7%, SD = 4.6%, Fig. 6c) compared to the conventional group with (M = 5.0%, SD = 5.8%, Fig. 6c).

In order to identify sex specific differences in the surgical outcome, ITI and BTB impingement severity, coverage percentage and third angle were compared (Table 2). It was identified that the impingement severity in men was significantly higher compared to the impingement severity in women for both ITI (p = 0.002) and BTB (p = 0.02) impingement. Although ITI coverage percentage was similar between men and women (p = 0.87), the BTB coverage percentage was significantly higher in women than in man (p = 0.05). In contrast, PO-ROM alignment, as defined by third angle, was significantly lower in women than in men for ITI impingement (p = 0.01), whereas it was comparable between both sexes (p = 0.30) for BTB impingement.

**Table 2:** Sex specific differences in ITI and BTB impingement.

	ITI Impingement			BTB Impingement		
	Severity	Coverage Percentage	Third Angle	Severity BTB	Coverage Percentage	Severity BTB
Women	1.5 (3.0)	180.6 (15.5)	16.6 (8.5)	3.9 (5.8)	153.3 (19.7)	16.0 (7.4)
Men	2.9 (3.7)	180.1 (13.1)	21.0 (10.2)	5.0 (4.6)	148.0 (16.0)	18.1 (8.5)
p-value	0.002	0.87	0.01	0.02	0.05	0.30

## Discussion

The aim of this study was to assess whether an intraoperative navigation guided femur first technique improves the surgical outcome in minimally invasive THA compared to a conventionally implanted control group. In addition, consistencies in surgical outcome due to the sex specific differences were also explored. In order to describe the effectiveness of the surgical outcome, PO-ROM area was simulated using post-operative CT scan and range of motion modelling, and compared with the B-ROM area, which is essential to perform activities of daily living (ADLs)^6, 13^. Three parameters were defined to express the effectiveness of the surgical outcome for both ITI and BTB impingement – (a) coverage percentage, (b) third angle, and (c) impingement severity.

It was identified that the impingement severity was significantly reduced for both ITI and BTB impingement in the navigation guided group compared to the free hand control group. In literature, bony and/or prosthetic impingement was identified as a major source of increased polyethylene wear and dislocation after THA^10, 20^. Consequently, it introduced a major impact on patient dissatisfaction and early revision surgery^21^. Compliant component position of cup and stem strongly affected impingement free ROM^22^. However, previous studies showed that the visual estimation of implant position harbored a high risk of misinterpretation^23^. Furthermore, even the knowledge of intraoperative stem version was not sufficient to realise a combined anteversion technique, and thus, to prevent impingement without the use of a cup alignment guide^24^. Navigation was shown to enable accurate intraoperative measurement of cup and stem position^25^. The previous ROM analysis of this study group showed higher flexion and internal rotation for the navigation guided implanted group in relation to the control group^12^. A more detailed 3D measurements in this current secondary analysis provided a greater insight for improved understanding of impingement. The potential reason for the reduced impingement severity in the navigation group was the improved alignment of PO-ROM axis compared to the control group although the size of ROM area was comparable. This indicated that the navigation adjusted cup position might provide a better alignment of the PO-ROM axis in terms of ADLs.

Regarding sex specific variations in ROM, it was found that the males were more prone to impingement than females. This was valid for both ITI and BTB impingement. Whereas the higher impingement severity for ITI impingement in men seemed to be related to a worse ROM axis alignment, the higher impingement severity for BTB impingement was associated with a higher ROM area size. These differences might be due to the functional differences like pelvic position as well as geometric differences of pelvic anatomy.

There are several limitation of this study. First, the current analysis focused on simulated PO-ROM analysis, and therefore, did not account for patient related functional outcome or implant longevity. Future analysis and long term follow up results of the study group should be carried out to prove the clinical relevance. Second, 3D-CT study allowed analysing ITI and BTB impingement without considering soft tissue restrictions, which could limit the ROM, especially in the obese patients. Third, functional parameters, such as pelvic tilt, were not included in the measurement of the current study. However, the issue of pelvic tilt is still an open question as pelvic tilt differs during gait cycle^26^, from preoperatively to postoperatively^27^, and from sitting to standing^28^. The use of navigation has four general limitations. Firstly, pelvic landmarks can become obscured by overlying soft-tissue (especially in the obese patients), which can make direct referencing for computer-assisted surgery difficult^15, 29^. Secondly, computers are susceptible to electronic failure, which happened once during the study. Therefore, surgeons, who are using navigation, should always be aware of potential malfunctions in the system, and should be able to continue operating without the assistance of a computer at any time. Thirdly, navigation systems and their service are expensive. Finally, both the registration and intra-operative measurement process of navigated THA significantly increase the operating time. In the study, this was increased by approximately ten minutes per operation. However, one of the strength in the study is the use of a single manufacturer’s THA design, which minimizes confounding factors. Any difference with regard to impingement is due to the operative technique only, rather than the prosthetic design of the components.

## Conclusions

The study focuses on to explore the effectivenss of navigated femur first surgical technique compared to the conventional technique. It was concluded from the study that the minimally invasive navigation guided femur first THA provides the possibility to reduce both ITI and BTB impingement in THA due to its improved alignment of post-operative-ROM axis with respect to the benchmark ROM axis. In addition, it was observed that men were more prone to impingement, and therefore, orthopedic surgeons should especially be aware of impingement in men while operating. Studies including long term results and functional aspects are required to prove the clinical relvance of the current data.
